# EXPLORING ABUSE ACROSS THE LIFE COURSE IN DEMENTIA CAREGIVING RELATIONSHIPS

**DOI:** 10.1093/geroni/igae098.0577

**Published:** 2024-12-31

**Authors:** Elizabeth Avent, Jeanine Yonashiro-Cho, Laura Mosqueda, Zachary Gassoumis

**Affiliations:** University of Chicago, Chicago, Illinois, United States; University of Southern California Keck School of Medicine, Los Angeles, California, United States; University of Southern California Keck School of Medicine, Pasadena, California, United States; University of Southern California, Los Angeles, California, United States

## Abstract

Based on the web of violence framework, described as different patterns of co-occurring abuse and trauma across the lifespan, caregivers who have lifespan patterns of abuse may be at greater risk for experiencing or perpetrating abuse once caregiving obligations begin. Conflict and abuse are concerns when the care receiver has ADRD, as behavioral problems associated with dementia may arise during caregiving. Spousal and intimate partner (S/IP) caregiving dyads are most likely to live together, so it is probable that aggression and abusive behaviors can be present in these dyads. Bivariate analyses (chi-square and t-tests) from surveys (N=207) and semi-structured interviews (N=12) with caregivers were conducted to identify characteristics that are conceptually related to abusive behavior throughout their relationships and life courses. There were no significant differences between S/IP caregivers and other types of caregiving relationships in mean adverse childhood experiences (ACE) scores, anxiety and depression scores, relationship quality, and caregiver burden. Roughly 22% of S/IP caregivers experienced at least 1 ACE. S/IP caregivers reported a history of IPV (10.9%) and abuse in the past year (12.2%). Among dyads in which recent abuse occurred, abuse was present throughout the relationship, even before onset of dementia. Semi-structured interviews showed that the caregiver experienced IPV in a previous relationship and/or at least one partner in the dyad experienced some form of family violence during childhood. Clarifying older victims’ experiences can help to identify risk factors, develop age-inclusive resources and services, and encourage collaboration between aging and domestic violence service providers.

